# Primary care clinicians’ opinions before and after implementation of cancer screening and prevention clinical decision support in a clinic cluster-randomized control trial: a survey research study

**DOI:** 10.1186/s12913-021-07421-0

**Published:** 2022-01-06

**Authors:** Melissa L. Harry, Ella A. Chrenka, Laura A. Freitag, Daniel M. Saman, Clayton I. Allen, Stephen E. Asche, Anjali R. Truitt, Heidi L. Ekstrom, Patrick J. O’Connor, Jo Ann M. Sperl-Hillen, Jeanette Y. Ziegenfuss, Thomas E. Elliott

**Affiliations:** 1 Essentia Institute of Rural Health, 502 E. Second Street, Duluth, MN 55805 USA; 2 HealthPartners Institute, 3311 E. Old Shakopee Road, Bloomington, MN 55425 USA; 3 Carle Foundation Hospital, Clinical Business and Intelligence, 611 W Park St, Urbana, IL 61801 USA

**Keywords:** Cancer prevention, Cancer screening, Clinical decision support, Electronic health record, Primary care provide

## Abstract

**Background:**

Electronic health record (EHR)-linked clinical decision support (CDS) may impact primary care clinicians’ (PCCs’) clinical care opinions. As part of a clinic cluster-randomized control trial (RCT) testing a cancer prevention and screening CDS system with patient and PCC printouts (with or without shared decision-making tools [SDMT]) for patients due for breast, cervical, colorectal, and lung cancer screening and/or human papillomavirus (HPV) vaccination compared to usual care (UC), we surveyed PCCs at study clinics pre- and post-CDS implementation. Our primary aim was to learn if PCCs' opinions changed over time within study arms. Secondary aims including examining whether PCCs' opinions in study arms differed both pre- and post-implementation, and gauging PCCs’ opinions on the CDS in the two intervention arms.

**Methods:**

This study was conducted within a healthcare system serving an upper Midwestern population. We administered pre-implementation (11/2/2017–1/24/2018) and post-implementation (2/2/2020–4/9/2020) cross-sectional electronic surveys to PCCs practicing within a RCT arm: UC; CDS; or CDS + SDMT. Bivariate analyses compared responses between study arms at both time periods and longitudinally within study arms.

**Results:**

Pre-implementation (53%, *n* = 166) and post-implementation (57%, *n* = 172) response rates were similar. No significant differences in PCC responses were seen between study arms on cancer prevention and screening questions pre-implementation, with few significant differences found between study arms post-implementation. However, significantly fewer intervention arm clinic PCCs reported being very comfortable with discussing breast cancer screening options with patients compared to UC post-implementation, as well as compared to the same intervention arms pre-implementation. Other significant differences were noted within arms longitudinally. For intervention arms, these differences related to CDS areas like EHR alerts, risk calculators, and ordering screening. Most intervention arm PCCs noted the CDS provided overdue screening alerts to which they were unaware. Few PCCs reported using the CDS, but most would recommend it to colleagues, expressed high CDS satisfaction rates, and thought patients liked the CDS’s information and utility.

**Conclusions:**

While appreciated by PCCs with high satisfaction rates, the CDS may lower PCCs’ confidence regarding discussing patients’ breast cancer screening options and may be used irregularly. Future research will evaluate the impact of the CDS on cancer prevention and screening rates.

**Trial registration:**

clinicaltrials.gov, NCT02986230, December 6, 2016.

## Background

Primary care clinicians (PCCs) manage multiple complex medical issues for a wide variety of patients, as well as must stay on top of patients’ preventative health care [1–9]. Given competing priorities, limited visit time, and the areas of importance for patients, cancer prevention and screening may be overlooked by both patients and PCCs [1, 4, 6–8, 10, 11]. Workable solutions are needed that adapt into clinic workflow, such as algorithm-based clinical decision support (CDS) systems connected with the electronic health record (EHR) [1, 2, 4, 6–8]. CDS can be used to help facilitate health care and save PCCs time and effort by: identifying numerous health care needs through instantly reviewing the EHR; alerting the user to context-specific knowledge; and assisting with medical decision-making [12–14]. A randomized control trial (RCT) of a web-based, patient-tailored, and EHR-linked CDS system, called “Priority Wizard” [15], targeted patients at risk for cardiovascular disease and showed a positive effect on both patients and clinicians by enhancing chronic disease health care for high-risk patients [15–18]. This CDS was updated to include targeted primary (human papillomavirus [HPV] vaccination, tobacco use, obesity) and secondary (breast, cervical, colorectal, lung) cancer prevention. Breast, cervical, colorectal, and lung cancer screening recommendations followed the United States Preventive Services Task Force (USPSTF) [19–22], and HPV vaccination recommendations followed the Centers for Disease Control and Prevention’s (CDC) Advisory Committee on Immunization Practices (ACIP) [23]. The CDS was designed to encompass multiple domains and the decision support component of the Chronic Care Model [24–26]. However, it was unknown whether expanding the CDS to include cancer prevention and screening might impact PCCs’ opinions on the specific cancer prevention and screening areas included in the CDS.

The objective of this exploratory study was to understand how the cancer prevention and screening CDS impacted PCCs’ views in a three arm, clinic cluster-RCT. One intervention arm implemented the CDS with cancer prevention and screening (breast, cervical, colorectal, and lung cancer and HPV vaccination) (CDS arm), the other intervention arm implemented the CDS cancer prevention and screening components with the addition of shared decision-making tools (SDMT) for breast, colorectal, and lung cancer screening and HPV vaccination (CDS + SDMT arm), and the third arm employed the usual care (UC) cancer prevention and screening patients received within the healthcare system (UC arm) [1, 2, 4, 6–8]. The primary aim of this study was to examine differences in PCC opinions within study arms between pre- and post-intervention implementation. Secondary aims included: 1) comparing PCC responses between study arms both pre- and post-intervention implementation; and 2) gauging PCC’s views of the cancer prevention and screening CDS components within the two intervention arms of the study post-implementation.

## Methods

### Sampling

The sample for the pre-implementation (*n =* 335) and post-implementation (*n* = 302) survey included PCCs that either worked 50% or more as a PCC or provided ongoing care for 25 or more eligible patients within one integrated healthcare system [4]. The healthcare system has primary care clinics located within three states in the Upper Midwest and serves a patient population that is predominantly rural [1, 2, 4, 6–8]. The study sample included advanced practitioners (nurse practitioners and physician assistants) and physicians (family practice or internal medicine) who practiced in at least one of 36 (including three clinics randomized together due to shared PCCs for 34 clinic randomization units) primary care or internal medicine clinics that were also part of the RCT assessing the impact of cancer prevention and screening CDS and SDMT [4]. Clinics were randomized in a 1:1:1 ratio to either the UC control arm that utilized the healthcare system’s typical cancer prevention and screening (*n* = 12), or to one of two intervention arms: either the CDS arm (*n* = 11), which received the CDS with cancer prevention and screening, or the CDS + SDMT arm (n = 11), which received both the CDS with cancer prevention and screening and SDMT for breast, colorectal, and lung cancer and HPV vaccination [8]. Detailed information on the design of the RCT is available in a paper by Elliott et al. [8], with final results of the RCT forthcoming. All clinics are part of a single healthcare system that uses the same cancer and prevention screening metrics systemwide within a single EHR (Epic^®^) [8].

### Survey instrument

Questions in both the pre- and post-intervention surveys were developed by the study team or adapted from the System Usability Scale (SUS) and the National Survey of Primary Care Physicians’ Recommendations & Practice for Breast, Cervical, Colorectal, & Lung Cancer Screening [4, 27, 28]. The post-implementation survey instrument included select questions previously reported in the pre-implementation survey sent to clinic PCCs 2 years previously [4]. These survey questions, presented in Tables 1, 2, 3, 4 and 5 of this paper, focused on: patient demographics; breast, cervical, colorectal, and lung cancer prevention and screening and HPV vaccination; the EHR; and shared decision-making between patients and PCCs [4]. The post-implementation survey instrument also included questions developed by the study team on the cancer prevention and screening CDS for PCCs working in the two intervention arm (CDS and CDS + SDMT) clinics.

**Table 1:** Respondent demographics by survey and study arm

	Pre-implementation survey					Post-implementation survey				
Survey Questions	*n*	UC *n* (%)	CDS *n* (%)	CDS + SDMT *n* (%)	*p*	*n*	UC *n* (%)	CDS *n* (%)	CDS + SDMT *n* (%)	*p*
Age Range (years)	164				0.129	165				0.449
≤ 34		15 (25.0)	11 (19.0)	8 (17.4)			14 (22.2)	9 (18.0)	16 (30.8)	
35–44		12 (20.0)	19 (32.8)	7 (15.2)			17 (27.0)	18 (36.0)	9 (17.3)	
45–54		17 (28.3)	10 (17.2)	8 (17.4)			14 (22.2)	11 (22.0)	15 (28.9)	
55–64		10 (16.7)	15 (25.9)	17 (37.0)			13 (20.6)	11 (22.0)	9 (17.3)	
≥ 65		6 (10.0)	3 (5.2)	6 (13.0)			5 (7.9)	1 (2.0)	3 (5.8)	
Pre-post within arm *p*^a^		0.809^b^	0.892	0.089						
Current Role	165				0.022	166				0.009
Nurse Practitioner		17 (28.3)	26 (44.8)	9 (19.2)			15 (23.8)	26 (51.0)	13 (25.0)	
Physician Assistant		6 (10.0)	1 (1.7)	9 (19.2)			11 (17.5)	2 (3.9)	11 (21.2)	
Family Practice physician		27 (45.0)	26 (44.8)	22 (46.8)			30 (47.6)	18 (35.3)	23 (44.2)	
Internal medicine physician		9 (15.0)	5 (8.6)	6 (12.8)			7 (11.1)	3 (5.9)	5 (9.6)	
Other		1 (1.7)	0 (0.0)	1 (2.1)			0 (0.0)	2 (3.9)	0 (0.0)	
Pre-post within arm *p*^a^		0.609	0.503	0.853						
Years in practice	165				0.020^b^	166				0.540^b^
≤ 5		24 (40.0)	19 (32.8)	10 (21.3)			21 (33.3)	16 (31.4)	23 (44.2)	
6–10		5 (8.3)	14 (24.1)	5 (10.6)			12 (19.1)	11 (21.6)	6 (11.5)	
≥ 11		31 (51.7)	25 (43.1)	32 (68.1)			30 (47.6)	24 (47.1)	23 (44.2)	
Pre-post within arm *p*^a^		0.220^b^	0.910^b^	0.040						
Days/week seeing patients	165				0.015	166				0.083
0–2		3 (5.0)	4 (6.9)	11 (23.4)			0 (0.0)	3 (5.9)	4 (7.7)	
3		13 (21.7)	9 (15.5)	4 (8.5)			11 (17.5)	11 (21.6)	6 (11.5)	
4		20 (33.3)	27 (46.6)	22 (46.8)			22 (34.9)	17 (33.3)	26 (50.0)	
5		24 (40.0)	18 (31.0)	10 (21.3)			30 (47.6)	20 (39.2)	16 (30.8)	
Pre-post within arm *p*^a^		0.329	0.521	0.169						
Sex	165				0.814^b^	166				0.925^b^
Female		39 (65.0)	35 (60.3)	28 (59.6)			40 (64.5)	34 (68.0)	34 (65.4)	
Male		21 (35.0)	23 (39.7)	19 (40.4)			22 (35.5)	16 (32.0)	18 (34.6)	
Pre-post within arm *p*^a^		0.955^b^	0.409^b^	0.551^b^						

Note. Freeman-Halton Test

*CDS* Clinical Decision Support, *SDMT* Shared Decision-Making Tool, *UC* Usual Care

^a^Comparing pre- and post-implementation survey responses within study arms

^b^Chi-square

**Table 2:** Prevention and screening preparedness, priority, comfort, recommendations, and risk calculators by survey and study arm

	Pre-implementation survey					Post-implementation survey				
Survey Questions	*n*	UC *n* (%)	CDS *n* (%)	CDS + SDMT *n* (%)	*p*	*n*	UC *n* (%)	CDS *n* (%)	CDS + SDMT *n* (%)	*p*
How well prepared do you feel to prioritize cancer risk factors and screening and discuss them with your patients?										
Not Prepared	164	1 (1.7)	1 (1.7)	2 (4.3)	0.891	172	0 (0.0)	0 (0.0)	0 (0.0)	0.056
Somewhat Prepared		22 (37.3)	19 (32.8)	16 (34.0)			14 (21.2)	22 (41.5)	17 (32.1)	
Very Prepared		36 (61.0)	38 (65.5)	29 (61.7)			52 (78.8)	31 (58.5)	36 (67.9)	
Pre-post within arm *p*^a^		0.038	0.431	0.392						
In relation to other health issues, what level of priority do your patients give cancer screening?										
Low Priority	165	4 (6.7)	2 (3.5)	0 (0.0)	0.361	170	2 (3.1)	4 (7.7)	1 (1.9)	0.669
Medium Priority		28 (46.7)	22 (37.9)	21 (44.7)			34 (52.3)	24 (46.2)	26 (49.1)	
High Priority		28 (46.7)	34 (58.6)	26 (55.3)			29 (44.6)	24 (46.2)	26 (49.1)	
Pre-post within arm *p*^a^		0.598	0.376	0.760						
How comfortable are you advising your patients on breast cancer screening options?										
Very uncomfortable	155	0 (0.0)	0 (0.0)	1 (2.3)	0.754	171	0 (0.0)	0 (0.0)	0 (0.0)	0.038
Somewhat uncomfortable		1 (1.8)	0 (0.0)	1 (2.3)			1 (1.5)	0 (0.0)	4 (7.6)	
Somewhat comfortable		13 (23.2)	16 (28.6)	10 (23.3)			24 (36.4)	27 (51.9)	27 (50.9)	
Very comfortable		42 (75.0)	40 (71.4)	31 (72.1)			41 (62.1)	25 (48.1)	22 (41.5)	
Pre-post within arm *p*^a^		0.240	0.013	0.004						
How comfortable are you advising patients about selecting a particular method of colorectal cancer screening?										
Very uncomfortable	153	0 (0.0)	0 (0.0)	0 (0.0)	0.089	167	0 (0.0)	0 (0.0)	1 (1.9)	0.434
Somewhat uncomfortable		0 (0.0)	0 (0.0)	1 (2.4)			1 (1.6)	1 (1.9)	1 (1.9)	
Somewhat comfortable		14 (25.5)	8 (14.3)	4 (9.5)			8 (12.7)	12 (23.1)	12 (23.1)	
Very comfortable		41 (74.6)	48 (85.7)	37 (88.1)			54 (85.7)	39 (75.0)	38 (73.1)	
Pre-post within arm *p*^a^		0.098	0.268	0.169						
How comfortable are you advising patients about selecting a particular method of lung cancer screening?										
Very uncomfortable	145	0 (0.0)	0 (0.0)	1 (2.6)	0.783	166	0 (0.0)	1 (2.0)	0 (0.0)	0.213
Somewhat uncomfortable		4 (7.7)	4 (7.4)	5 (12.8)			3 (4.8)	7 (13.7)	5 (9.6)	
Somewhat comfortable		21 (40.4)	24 (44.4)	16 (41.0)			22 (34.9)	22 (43.1)	17 (32.7)	
Very comfortable		27 (51.9)	26 (48.2)	17 (43.6)			38 (60.3)	21 (41.2)	30 (57.7)	
Pre-post within arm *p*^a^		0.625	0.518	0.433						
How often did you recommend colorectal cancer screening tests other than colonoscopy to your asymptomatic, average-risk patients during the past 12 months?										
Never	153	2 (3.6)	1 (1.8)	1 (2.4)	0.806	168	0 (0.0)	3 (5.8)	2 (3.9)	0.349
Sometimes		23 (41.8)	28 (50.0)	19 (45.2)			21 (32.8)	20 (38.5)	18 (34.6)	
Usually		12 (21.8)	14 (25.0)	7 (16.7)			17 (26.6)	16 (30.8)	17 (32.7)	
Always		18 (32.7)	13 (23.2)	15 (35.7)			26 (40.6)	13 (25.0)	15 (28.9)	
Pre-post within arm *p*^a^		0.338	0.521	0.330						
At preventive care visits, how often do you use a cancer risk calculation for any of the following?										
Breast cancer (e.g., BCRAT)										
Never	154	44 (80.0)	43 (76.8)	37 (86.1)	0.225	171	44 (67.7)	39 (73.6)	34 (65.4)	0.855
Sometimes		9 (16.4)	10 (17.9)	2 (4.7)			15 (22.7)	9 (17.0)	14 (26.9)	
Usually		2 (3.6)	1 (1.8)	2 (4.7)			5 (7.6)	4 (7.6)	2 (3.9)	
Always		0 (0.0)	2 (3.6)	2 (4.7)			2 (3.0)	1 (1.9)	2 (3.9)	
Pre-post within arm *p*^a^		0.328	0.569	0.017						
Colorectal cancer										
Never	153	48 (87.3)	46 (82.1)	36 (85.7)	0.745	169	56 (84.9)	42 (82.4)	35 (67.3)	0.161
Sometimes		5 (9.1)	7 (12.5)	3 (7.1)			6 (9.1)	4 (7.8)	12 (23.1)	
Usually		2 (3.6)	1 (1.8)	1 (2.4)			2 (3.0)	4 (7.8)	4 (7.7)	
Always		0 (0.0)	2 (3.6)	2 (4.8)			2 (3.0)	1 (2.0)	1 (1.9)	
Pre-post within arm *p*^a^		0.778	0.440	0.080						
Lung cancer (e.g., CDC/AHRQ)										
Never	154	42 (76.4)	43 (76.8)	35 (81.4)	0.936	164	46 (74.2)	35 (68.6)	25 (49.0)	0.184
Sometimes		7 (12.7)	7 (12.5)	5 (11.6)			9 (14.5)	9 (17.7)	15 (29.4)	
Usually		5 (9.1)	3 (5.4)	2 (4.7)			5 (8.1)	4 (7.8)	6 (11.8)	
Always		1 (1.8)	3 (5.4)	1 (2.3)			2 (3.2)	3 (5.9)	5 (9.8)	
Pre-post within arm *p*^a^		0.979	0.783	0.012						

Note. Freeman-Halton Exact Test. *CDS* Clinical Decision Support, *SDMT* Shared Decision-Making Tool, *UC* Usual Care

^a^Comparing pre- and post-implementation survey responses within study arms

**Table 3:** Colorectal and lung cancer prevention and screening discussions and decision-making by survey and study arm

	Pre-Implementation Survey					Post-Implementation Survey				
Survey Questions	n	UC *n* (%)	CDS *n* (%)	CDS + SDMT *n* (%)	*p*	n	UC *n* (%)	CDS *n* (%)	CDS + SDMT *n* (%)	*p*
Consider the last patient you saw regarding colorectal cancer screening. For each of the following six statements related to the decision-making in this consultation, please select the response that best describes your actions with this patient.										
I told my patient that there are different screening options (e.g. FIT, Colonoscopy)										
Agree	152	49 (89.1)	49 (87.5)	39 (95.1)	0.838	168	56 (87.5)	45 (86.5)	47 (90.4)	0.409
Neither		1 (1.8)	1 (1.8)	0 (0.0)			0 (0)	1 (1.9)	2 (3.9)	
Disagree		5 (9.1)	6 (10.7)	2 (4.9)			8 (12.5)	6 (11.5)	3 (5.8)	
Pre-post within arm *p*^a^		0.559	1.000	0.691						
I explained the advantage and disadvantages of the screening options to my patient										
Agree	152	49 (89.1)	49 (87.5)	40 (97.6)	0.489	167	56 (87.5)	45 (86.5)	46 (90.2)	0.411
Neither		1 (1.8)	2 (3.6)	0 (0.0)			0 (0)	1 (1.9)	2 (3.9)	
Disagree		5 (9.1)	5 (8.9)	1 (2.4)			8 (12.5)	6 (11.5)	3 (5.9)	
Pre-post within arm *p*^a^		0.559	0.905	0.448						
I helped my patient understand all the information										
Agree	150	49 (90.7)	47 (85.5)	40 (97.6)	0.442	166	54 (87.1)	45 (86.5)	46 (88.5)	0.601
Neither		2 (3.7)	3 (5.5)	0 (0.0)			1 (1.6)	1 (1.9)	3 (5.8)	
Disagree		3 (5.6)	5 (9.1)	1 (2.4)			7 (11.3)	6 (11.5)	3 (5.8)	
Pre-post within arm *p*^a^		0.440	0.688	0.284						
I asked my patient which screening option he/she prefers										
Agree	152	47 (85.5)	50 (89.3)	38 (92.7)	0.837	168	53 (82.8)	43 (82.7)	47 (90.4)	0.152
Neither		2 (3.6)	1 (1.8)	1 (2.4)			0 (0)	2 (3.9)	2 (3.9)	
Disagree		6 (10.9)	5 (8.9)	2 (4.9)			11 (17.2)	7 (13.5)	3 (5.8)	
Pre-post within arm *p*^a^		0.206	0.583	1.000						
My patient and I selected a screening option together										
Agree	150	43 (78.2)	49 (87.5)	38 (97.4)	0.054	168	53 (82.8)	45 (86.5)	46 (88.5)	0.302
Neither		7 (12.7)	2 (3.6)	0 (0.0)			0 (0)	1 (1.9)	2 (3.9)	
Disagree		5 (9.1)	5 (8.9)	1 (2.6)			11 (17.2)	6 (11.5)	4 (7.7)	
Pre-post within arm *p*^a^		0.005	0.905	0.383						
My patient and I reached an agreement on how to proceed										
Agree	150	46 (83.6)	46 (85.2)	38 (92.7)	0.522	167	54 (85.7)	45 (86.5)	47 (90.4)	0.324
Neither		5 (9.1)	2 (3.7)	1 (2.4)			0 (0)	1 (1.9)	2 (3.8)	
Disagree		4 (7.3)	6 (11.1)	2 (4.9)			9 (14.3)	6 (11.5)	3 (5.8)	
Pre-post within arm *p*^a^		0.025	1.000	1.000						
Consider the last patient you saw who was a possible candidate for lung cancer screening. For each of the following two statements related to the decision-making in this consultation, please select the response that best describes your actions with this patient.										
I helped my patient understand all the risks and benefits and details about screening										
Agree	146	37 (69.8)	35 (64.8)	28 (71.8)	0.665	165	53 (84.1)	40 (78.4)	43 (84.3)	0.030
Neither		6 (11.3)	11 (20.4)	4 (10.3)			2 (3.2)	9 (17.7)	2 (3.9)	
Disagree		10 (18.9)	8 (14.8)	7 (18.0)			8 (12.7)	2 (3.9)	6 (11.8)	
Pre-post within arm *p*^a^		0.122	0.136	0.305						
My patient and I reached an agreement on how to proceed										
Agree	142	37 (69.8)	34 (65.4)	26 (70.3)	0.957	159	50 (84.8)	39 (79.6)	44 (86.3)	0.002
Neither		9 (17.0)	10 (19.2)	5 (13.5)			1 (1.7)	9 (18.4)	1 (2.0)	
Disagree		7 (13.2)	8 (15.4)	6 (16.2)			8 (13.6)	1 (2.0)	6 (11.8)	
Pre-post within arm *p*^a^		0.015	0.060	0.076						

Note. Freeman-Halton Exact Test. *CDS* Clinical Decision Support, *SDMT* Shared Decision-Making Tool, *UC* Usual Care

^a^Comparing pre- and post-implementation survey responses within study arms

**Table 4:** Provider perspectives on the EMR for cancer prevention and screening by survey and study arm

	Pre-Implementation Survey					Post-Implementation Survey				
Survey Questions	*n*	UC *n* (%)	CDS *n* (%)	CDS + SDMT *n* (%)	*p*	*n*	UC *n* (%)	CDS *n* (%)	CDS + SDMT *n* (%)	*p*
Select “yes” or “no” for the following questions on the EMR your clinic uses for screening of breast, cervical, colorectal, or lung cancers or HPV vaccination.										
The EMR alerts me that this action is due.										
Breast Cancer Screening	156	56 (96.6)	50 (90.9)	40 (93.0)	0.506^a^	171	66 (100)	50 (96.2)	51 (96.2)	0.247^a^
Pre-post within arm *p*^b^		0.217^a^	0.439^a^	0.654^a^						
Cervical Cancer Screening	155	20 (35.1)	18 (32.7)	12 (27.9)	0.745	172	29 (43.9)	29 (54.7)	23 (43.4)	0.409
Pre-post within arm *p*^b^		0.317	0.021	0.117						
Colorectal Screening	153	54 (94.7)	49 (90.7)	36 (85.7)	0.310^a^	172	64 (97.0)	48 (90.6)	49 (92.5)	0.380^a^
Pre-post within arm *p*^b^		0.662^a^	0.975	0.329^a^						
Lung Screening	153	41 (73.2)	35 (63.6)	21 (50.0)	0.062	169	56 (86.2)	43 (84.3)	40 (75.5)	0.287
Pre-post within arm *p*^b^		0.075	0.016	0.010						
HPV Vaccination	153	39 (70.9)	44 (80.0)	30 (69.8)	0.429	168	41 (65.1)	37 (71.2)	37 (69.8)	0.758
Pre-post within arm *p*^b^		0.499	0.286	0.996						
The EMR makes it easy for me to order the needed service.										
Breast Cancer Screening	154	49 (87.5)	44 (80.0)	37 (86.1)	0.520	171	58 (87.9)	42 (80.8)	46 (86.8)	0.522
Pre-post within arm *p*^b^		0.949	0.920	0.915						
Cervical Cancer Screening	153	30 (53.6)	34 (61.8)	28 (66.7)	0.403	170	44 (67.7)	34 (65.4)	36 (67.9)	0.953
Pre-post within arm *p*^b^		0.112	0.702	0.897						
Colorectal Screening	153	51 (91.1)	40 (74.1)	37 (86.1)	0.048	172	63 (95.5)	45 (84.9)	47 (88.7)	0.130^a^
Pre-post within arm *p*^b^		0.468^a^	0.166	0.698						
Lung Screening	149	42 (75.0)	30 (56.6)	26 (65.0)	0.128	172	57 (86.4)	40 (75.5)	41 (77.4)	0.273
Pre-post within arm *p*^b^		0.110	0.043	0.189						
HPV Vaccination	148	40 (74.1)	43 (82.7)	30 (71.4)	0.391	167	44 (69.8)	35 (68.6)	36 (67.9)	0.975
Pre-post within arm *p*^b^		0.612	0.960	0.713						
The EMR enables me to print out materials that help patients identify their preferred screening method.										
Breast Cancer Screening	151	18 (32.7)	20 (37.0)	11 (26.2)	0.530	165	17 (26.2)	25 (50.0)	20 (40.0)	0.030
Pre-post within arm *p*^b^		0.430	0.183	0.163						
Cervical Cancer Screening	149	16 (29.1)	17 (32.1)	9 (22.0)	0.547	165	16 (24.6)	25 (50.0)	16 (32.0)	0.016
Pre-post within arm *p*^b^		0.581	0.064	0.285						
Colorectal Screening	148	19 (34.6)	22 (43.1)	14 (33.3)	0.548	165	18 (27.7)	26 (52.0)	20 (40.0)	0.029
Pre-post within arm *p*^b^		0.418	0.373	0.509						
Lung Screening	148	18 (33.3)	18 (34.0)	11 (26.8)	0.726	163	23 (35.9)	26 (52.0)	20 (40.8)	0.220
Pre-post within arm *p*^b^		0.767	0.064	0.164						
HPV Vaccination	145	20 (38.5)	21 (40.4)	10 (24.4)	0.228	160	17 (26.6)	23 (48.9)	20 (40.8)	0.047
Pre-post within arm *p*^b^		0.172	0.393	0.100						
The EMR makes it easy to calculate cancer risks for individual patients.										
Breast Cancer Screening	152	4 (7.1)	5 (9.1)	3 (7.3)	0.932^a^	170	8 (12.1)	11 (21.6)	12 (22.6)	0.256
Pre-post within arm *p*^b^		0.544^a^	0.073	0.051^a^						
Cervical Cancer Screening	153	5 (8.9)	4 (7.3)	2 (4.8)	0.861^a^	170	5 (7.7)	9 (17.3)	9 (17.0)	0.216
Pre-post within arm *p*^b^		0.806	0.144^a^	0.105^a^						
Colorectal Screening	153	6 (10.7)	6 (10.9)	4 (9.5)	1.000^a^	168	5 (7.7)	8 (15.4)	13 (25.5)	0.031
Pre-post within arm *p*^b^		0.564	0.493	0.061^a^						
Lung Screening	151	14 (25.5)	9 (16.4)	6 (14.6)	0.329^a^	169	15 (22.7)	13 (25.5)	20 (38.5)	0.146
Pre-post within arm *p*^b^		0.726	0.247	0.011						
HPV Vaccination	152	10 (18.2)	6 (10.9)	3 (7.1)	0.262^a^	166	6 (9.2)	7 (14.3)	12 (23.1)	0.113
Pre-post within arm *p*^b^		0.151	0.603	0.048^a^						

Note. Chi-square. Yes responses shown. *CDS* Clinical Decision Support, *SDMT* Shared Decision-Making Tool, *UC* Usual Care

^a^Fisher’s Exact Test

^b^Comparing pre- and post-implementation survey responses within study arms

**Table 5:** Post-implementation survey: intervention clinic CDS questions and responses by study arm

Survey Questions	*n*	CDS *n* (%)				CDS + SDMT *n* (%)				
Please indicate the extent to which you agree or disagree with the following statements. The Wizard...		Agree	Neither	Disagree		Agree	Neither	Disagree		*p*
Helps get more patients screened for cancer	101	21 (41.2)	15 (29.4)	15 (29.4)		29 (58.0)	13 (26.0)	8 (16.0)		0.170
Saves me time talking to patients	101	6 (11.8)	18 (35.3)	27 (52.9)		11 (22.0)	20 (40.0)	19 (38.0)		0.228
Increases the appointment length	100	25 (50.0)	20 (40.0)	5 (10.0)		24 (48.0)	20 (40.0)	6 (12.0)		0.946
Is a useful tool for shared decision-making	99	24 (50.0)	17 (35.4)	7 (14.6)		31 (60.8)	15 (29.4)	5 (9.8)		0.533
Influences my treatment recommendations	101	14 (28.0)	20 (40.0)	16 (32.0)		28 (54.9)	14 (27.5)	9 (17.7)		0.022
Influences my patients’ decisions	102	13 (25.5)	23 (45.1)	15 (29.4)		23 (45.1)	20 (39.2)	8 (15.7)		0.077
Helps me place orders	101	11 (22.0)	18 (36.0)	21 (42.0)		13 (25.5)	18 (35.3)	20 (39.2)		0.913
Provides accurate information	100	23 (46.0)	21 (42.0)	6 (12.0)		30 (60.0)	15 (30.0)	5 (10.0)		0.365
How often do you … ..		Always	Usually	Sometimes	Never	Always	Usually	Sometimes	Never	*p*
Use the Wizard paper printouts^a^	102	3 (5.9)	6 (11.8)	23 (45.1)	19 (37.3)	3 (5.9)	7 (13.7)	24 (47.1)	17 (33.3)	0.975
Use the Wizard on-screen (*n* = 51/51)^a^	102	1 (2.0)	5 (9.8)	16 (31.4)	29 (56.9)	1 (2.0)	9 (17.7)	20 (39.2)	21 (41.2)	0.382
Use the tools within the Wizard’s Decision Aid tab (n = 51/48)^a^	99	0 (0)	3 (6.3)	15 (31.3)	30 (62.5)	1 (2.0)	5 (9.8)	22 (43.1)	23 (45.1)	0.274
		Yes definitely		Yes somewhat	No	Yes definitely	Yes somewhat		No	*p*
Do your patients like the Wizard’s information regarding cancer screening and prevention?^a^	92	0 (0)		32 (72.7)	12 (27.3)	12 (25.0)	23 (47.9)		13 (27.1)	< 0.001
Is there value in patients taking the Wizard information home with them?	95	5 (10.9)		32 (69.6)	9 (19.6)	17 (34.7)	20 (40.8)		12 (24.5)	0.008
Would you recommend the Wizard to colleagues?^a^	96	2 (4.4)		28 (60.9)	16 (34.8)	11 (22.0)	28 (56.0)		11 (22.0)	0.030
Does the Wizard alert you when a patient is overdue for a cancer screening you were NOT aware of?	97	8 (17.0)		19 (40.4)	20 (42.6)	11 (22.0)	23 (46.0)		16 (32.0)	0.547
		Very satisfied		Somewhat satisfied	Not at all satisfied	Very satisfied	Somewhat satisfied		Not at all satisfied	*p*
Regarding cancer screening and prevention, how satisfied are you with the Wizard?^a^	96	4 (8.5)		31 (66.0)	12 (25.5)	13 (26.5)	28 (57.1)		8 (16.3)	0.062

Note. The Wizard is the CDS. *CDS* Clinical Decision Support, *SDMT* Shared Decision-Making Tool

^a^Freeman-Halton exact test

### Intervention

Intervention arm clinics followed the same workflow as for the cardiovascular CDS studies [15]. In both intervention arms (CDS and CDS + SDMT), rooming staff measured and entered patients’ blood pressure into the EHR, which triggered the web-based CDS via an alert built into the EHR instructing the rooming staff to print two patient-tailored handouts for eligible patients: a patient version and a PCC version with more detailed information specific to the patient [1, 2, 4, 6–8]. In the CDS + SDMT arm, rooming staff also printed abbreviated SDMT for breast, colorectal, or lung cancer screening and HPV vaccination in patients due or overdue for these items [1, 2, 4, 6–8]. An electronic version of the CDS was available to PCCs practicing in CDS and CDS + SDMT study arms that included access to breast, colorectal, and lung cancer risk calculators and multi-page SDMT for breast, colorectal, and lung cancer screening and HPV vaccination in the CDS + SDMT study arm [1, 2, 6–8]. Rooming staff and PCCs in clinics randomized to the UC arm did not have access to the CDS or SDMT, and instead provided the same cancer prevention and screening care as other clinics in the healthcare system [ 1, 2, 4, 6–8]. This included electronic best practice alerts or flags in the EHR for patients due or overdue for breast, colon, and lung cancer screening or HPV vaccination. The EHR lacked a best practice alert for cervical cancer screening at the time of our study. All intervention arm clinics received baseline and booster training, including ongoing in person trainings conducted by study team members at intervention clinics, electronic self-completed training administered through the healthcare system’s employee training system that included a video tutorial, and distributed information on the CDS and SDMT to intervention clinic managers for further distribution to clinic staff [1, 2, 8]. In addition, we conducted virtual webinar trainings [2]. A feedback button was built into the CDS so that rooming staff and PCCs could alert the study team to any issues experienced with the CDS [1]. Study team members gave monthly updates to clinics that presented individualized reports of how well the clinic was doing reaching the recommended 80% CDS print rate (e.g., intervention clinics were encouraged to print the CDS, and any abbreviated SDMT in the CDS + SDMT arm, for approximately 80% of eligible patients) [8]. There was also a two-week suppression of the CDS for eligible patients to prevent alert fatigue [8].

### Data collection

Information on the pre-implementation survey, conducted from November 2, 2017 to January 24, 2018, has been previously published in a paper comparing responses between physicians and advanced practitioners [4]. The electronic post-implementation survey was administered through email from February 2, 2020 to April 9, 2020. First, a notification email signed by a healthcare system primary care leader was sent notifying all eligible PCCs of the upcoming survey and encouraging them to complete it. Next, an initial invitation email with a link to the survey was sent to PCCs, who were emailed up to 11 reminders over the course of 12 weeks linking them to the survey. Emails and surveys were administered using REDCap electronic data capture tools [29, 30]. Completion of either survey implied PCC consent. The healthcare system’s Institutional Review Board reviewed, approved, and monitored this survey and RCT study, and it was also monitored by an Indendent Project Safety Officer.

### Data analysis

Descriptive analyses and tests of association were conducted in SAS v. 9.4 and SAS Enterprise Guide v. 8.1 [31, 32]. Responses to each item were summarized by study arm (UC, CDS, or CDS + SDMT) and are included in Tables 1, 2, 3, 4 and 5. For items with five-tiered Likert scale responses (strongly disagree, somewhat disagree, neither agree or disagree, somewhat agree, strongly agree), responses were recoded into three levels (disagree, neither, agree) to allow for more straightforward interpretation. Chi-square statistics, Fisher’s exact tests (expected cell count < 5 in 2 × 2 tables), or Freeman-Halton tests (expected cell count < 5 in *R* × *C* tables) were used to determine significant differences in responses between the three arms and within arms temporally. Tests were two-tailed with an alpha of 0.05.

## Results

Pre- and post-implementation survey respondent demographics within each of the three study arms are presented in Table 1. Of the 335 pre-implementation surveys sent to 312 active email addresses, 165 were fully or partially completed by PCCs (53% response rate) [4]. Post-implementation surveys were administered to 302 PCCs with 301 having an active email address, and were fully or partially completed by 172 PCCs, resulting in a response rate of 57%. We found significant differences between the three arms related to PCCs’ current role at pre- (*p* = 0.022) and post-implementation (*p* = 0.009). Family practice physicians were the most common role represented in both surveys. The majority of respondents had 11 of more years in practice, and years in practice did differ significantly between study arms pre-implementation (*p* = 0.020), but not post-implementation (*p* = 0.540). The number of days per week PCCs saw patients also differed significantly between study arms pre-implementation (*p* = 0.015), but not post-implementation (*p* = 0.083). Most respondents saw patients 4–5 days a week in the clinic, and the majority of respondents were female in all study arms.

### Pre-implementation survey comparison between study arms

Statistical comparisons of cancer prevention and screening survey questions between the three study arms pre-implementation are presented in Tables 2, 3 and 4. No significant differences between study arms were seen on these questions prior to CDS implementation.

### Post-implementation survey comparison between study arms

#### Cancer screening and prevention

At post-implementation, there was a significant difference between study arms in how prepared PCCs felt to prioritize cancer risk factors and screening and to discuss them with patients. UC PCCs felt more prepared than the other two groups, although this did not reach the level of significance (*p* = 0.056), and the majority of respondent felt “very prepared” in all groups (Table 2). Cancer screening was ranked as either a medium or high priority for PCCs' patients in all arms, with only 4% (*n* = 7) of PCCs ranking screening as “low priority” for patients. UC clinic PCCs reported significantly higher rates of feeling very comfortable advising patients on breast cancer screening option a as compared to the intervention arms (62% vs. 48% and 42%, *p* = 0.038) (Table 2).

When asked to consider the last patient PCCs saw who was eligible for lung cancer screening, UC and CDS + SDMT respondents were significantly more likely to report not being able to present all options, risks, and benefits (*p* = 0.030) and ending their interaction with no agreement with the patient as to how to proceed (*p* = 0.002) than CDS arm respondents (Table 3). However, for these and other items in Table 3, PCCs gave generally affirmative responses when grading their actions within colorectal and lung cancer discussions with patients.

#### Electronic health record

Similar to pre-implementation, there were no significant differences between arms when it came to describing most EHR uses for breast, cervical, colorectal, or lung cancer screening or HPV vaccination post-implementation (Table 4). Almost all of the PCCs in the three arms reported that the EHR alerted them when breast cancer screening was due (UC = 100%, CDS = 96%, CDS + SDMT = 96%), while alerts for cervical cancer screening were least frequently reported (UC = 44%, CDS = 55%, CDS + SDMT = 43%). The groups all had a majority of PCCs agree that the EHR made it easy for them to order screening for all conditions. There was a significant difference in responses when it came to whether the EHR allowed PCCs to print materials to help patients identify their preferred screening method, with the CDS group reporting significantly more frequent use of this functionality for breast (UC = 26%, CDS = 50%, CDS + SDMT = 40%, *p* = 0.030), cervical (UC = 25%, CDS = 50%, CDS + SDMT = 32%, *p* = 0.016), and colorectal cancer (UC = 28%, CDS = 52%, CDS + SDMT = 40%, *p* = 0.029) and HPV vaccination (UC = 27%, CDS = 49%, CDS + SDMT = 41%, *p* = 0.047), but not for lung cancer (UC = 36%, CDS = 52%, CDS + SDMT = 41%, *p* = 0.220). Overall, PCCs reported that the EHR did not make it easy to calculate cancer risk for an individual patient, but the CDS + SDMT group agreed that the EHR allowed them to calculate colorectal cancer risks more easily for individual patients as compared to the other two arms (UC = 8%, CDS = 15%, CDS + SDMT = 26%) (*p* = 0.031).

#### CDS functionality and use in the CDS and CDS + SDMT intervention arms

Table 5 shows responses to questions pertaining to CDS functionality, which were only included on the surveys sent to PCCs at the two intervention arms post-implementation. Compared to the CDS arm, the CDS + SDMT arm PCCs were more likely to responded they definitely agreed that their patients liked (0% vs. 25%, *p* < 0.001) and valued the CDS’s information (11% vs. 35%, *p* = 0.008). Also, compared to the CDS arm, a significantly higher percentage of CDS + SDMT PCCs were more likely to recommend the CDS to colleagues (65% vs. 78%, *p* = 0.030) and agree that the CDS influenced their treatment recommendations (68% vs. 82%, *p* = 0.022). Both groups agreed that the CDS helped them get more patients screened for cancer, was useful for shared decision-making, and provided accurate information. While not significantly different (*p* = 0.228), the CDS arm PCCs were more likely to disagree (53%) that the CDS saved them time talking to patients than the CDS + SDMT arm PCCs (38%). PCCs’ levels of being very or somewhat satisfied with the CDS for cancer prevention and screening where high at 84% in the CDS + SDMT arm and 75% in the CDS arm, although this difference was not statistically significant (*p* = 0.062).

### Comparison between pre-implementation and post-implementation surveys

The respondents for both pre-implementation and post-implementation surveys were similar in their demographics, with family practice physicians the most common role represented, and the majority of respondents having 6 or more years in practice (Table 1). Compared to pre-implementation, there was an increase in PCCs responding that they felt very prepared to prioritize cancer risk factors and screening post-implementation (63–79%) (not shown). The CDS + SDMT and CDS arms reported being more likely to use the cancer risk calculation tool for breast, colorectal, and lung cancer as compared to the overall responses from the pre-implementation survey. The percent of respondents that ranked cancer screening as a high priority for patients fell overall from 53 to 45%, with none of the three arms responding at a higher rate. Similarly, there was a decrease in PCCs who responded that they were very comfortable advising their patients on breast cancer screening options (73–51%), although there was a slight increase in overall very comfortable responses for the same question regarding lung cancer screening (48–54%). There was no observed difference in reports of how PCC utilized the EHR for cancer screenings or in the reported frequency of recommending colorectal cancer screening tests other than colonoscopy to asymptomatic, average-risk patients (Always 30–32%).

#### Longitudinal within study arm comparisons

Some significant differences were seen when comparing responses on cancer prevention and screening items longitudinally within study arms (Tables 2, 3 and 4). In the UC arm, more PCCs (79%) reporting feeling very prepared to prioritize risk factors post-implementation compared to pre-implementation (61%) (*p* = 0.038) (Table 2). Post-implementation, more PCCs described either disagreeing (17%) or agreeing (83%) that they selected a colorectal cancer screening option together with patients than pre-implementation (9, 78%), with none responding “neither” post-implementation (*p* = 0.005) (Table 3). Similarly, no PCCs responded “neither” on reaching an agreement on how to proceed with colorectal cancer screening with patients post-implementation (*p* = 0.025), although rates of agreement were similar between pre-implementation (84%) and post-implementation (86%). Post-implementation, more PCCs (85%) affirmed that they reached an agreement on how to proceed with patients due or overdue for lung cancer screening than pre-implementation (70%) (*p* = 0.015).

Within the CDS intervention arm, the already high level of comfort with advising patients about breast cancer screening options declined slightly over time. Fewer PCCs describing being very comfortable post-implementation (48%), with more somewhat comfortable (52%) compared to pre-implementation (very comfortable = 71%, somewhat comfortable = 29%, *p* = 0.013) (Table 2). Significantly more post-implementation PCCs (55%) responded that the EHR alerted them when cervical cancer screening was due than pre-implementation (33%, *p* = 0.021) (Table 4). Similarly, more PCCs responded that the EHR alerted them to lung cancer screening post-implementation (84%) than pre-implementation (64%, *p* = 0.016). More PCCs also agreed that the EHR made it easy to order the needed service for lung cancer screening post-implementation (76%) than pre-implementation (57%, *p* = 0.043).

In the CDS + SDMT intervention arm, while PCCs still primarily responded as being very or somewhat comfortable advising patients on breast cancer screening options post-implementation, fewer reported being very comfortable (42%) than pre-implementation (72%, *p* = 0.004) (Table 2). Most respondents did not use risk calculators; however, fewer PCCs post-implementation reported never using a breast cancer risk calculator (65% vs. 86%, *p* = 0.017) or a lung cancer risk calculator (49% vs. 81%, *p* = 0.012) compared to pre-implementation. Significantly more PCCs answered that the EHR alerted them to patients due for lung cancer screening post-implementation (76%) than pre-implementation (50.0%, *p* = 0.010) (Table 4). Post-implementation, more PCCs noted the EHR made it easy to calculate patients’ lung cancer risk (39% vs. 15%, *p* = 0.011) and HPV risk (23% vs. 7%, *p* = 0.048) than pre-implementation, although rates were still low. No other significant differences were seen between study arms over time regarding post-implementation survey items.

## Discussion

In this study, we found no significant differences in PCCs’ opinions on cancer prevention and screening questions related to breast, cervical, colorectal, or lung cancer or HPV vaccination between the control arm receiving UC and two CDS-focused intervention arms in a clinic-RCT prior to CDS implementation. Some significant differences were seen between study arms post-CDS implementation, such as UC PCCs having higher rates of comfort discussing breast cancer screening options with patients than in both intervention arms, and CDS intervention arm PCCs having higher rates of noting the EHR printed breast, cervical, colorectal, and HPV vaccination materials and calculating colorectal cancer risk than UC PCCs. Furthermore, we found significant differences in survey responses within all three study arms over time, but these differed between UC and the two intervention arms. Significant differences in survey items within intervention arms were primarily in areas where the CDS and/or SDMT could be expected to impact clinical practice. These included significant increases in EHR alerts for patients overdue for cervical (CDS arm) and lung (CDS and CDS + SDMT arms) cancer screening, improving ordering of lung cancer screening in the EHR (CDS arm), and providing opportunities for lung and HPV risk calculation in the EHR (CDS + SDMT arm). Rates of using a lung or breast cancer risk calculator also increased significantly in at least one intervention arm (CDS + SDMT). Of note, both intervention arms saw significant decreases in how comfortable PCCs were in advising patients on available breast cancer screening options; however, rates stayed similar over time in the usual care arm. Most CDS and CDS + SDMT intervention arm PCCs found value in the CDS, would recommend it to colleagues, and thought it provided benefits to patients, but few PCCs reported regularly using the CDS.

A recent survey of 37 PCCs practicing in another Midwestern healthcare system found that most PCCs were comfortable using CDS system alerts for patients overdue for cancer prevention and screening, but that compared to physicians, advanced practice clinicians (e.g., nurse practitioners and physician assistants) had significantly higher rates of agreeing that CDS system alerts were straightforward, the current number of alerts was acceptable, and that more alerts were needed [33]. While the present study did not compare post-implementation responses by PCC type, we reported a similar comparison between PCC types pre-implementation [4], with few significant differences seen between physicians and advanced practice clinicians.

The findings from our post-CDS-implementation survey suggest modest impacts of the CDS on PCC opinions related to the EHR and some cancer prevention and screening areas. Yet the low rate of self-reported CDS use is a concern as it may allude to issues with intervention adoption. Other studies have also demonstrated many barriers to clinician uptake of a CDS tool. Workflow incompatibilities, redundant alerts, time and resource burden, incorrect material, not being appropriate for the situation, repetitive information, limited training, and feeling threatened by the technology were all cited by clinicians as barriers to using CDS [34, 35]. As we noted previously [2], the “Ten Commandments for Effective Clinical Decision Support” described by Bates et al. in 2003 are still relevant today, particularly those related to CDS and time, workflow, hard stops, simplicity, conciseness, monitoring and responding, and managing the CDS systems [36]. The GUIDES checklist, published after our CDS was developed, appears to be a useful tool to aid adoption and use of future cancer prevention and screening-focused CDS in primary care [2, 37].

Of note, PCCs in both intervention arms reported significantly lower levels of being very comfortable regarding making breast cancer screening decisions with patients post-implementation compared to pre-implementation. This may be due to the CDS and the SDMT using USPSTF recommendations for breast cancer screening for average risk women (mammogram every other year starting at age 50) [19] that differed from the healthcare system’s recommendations that all women age 40 and older receive annual mammograms (similar to recommendations by the American College of Radiology) [38, 39]. The CDS and SDMT also recommended that PCCs calculate patients’ breast cancer risk score in making shared decisions with patients regarding when to start screening for breast cancer in individual patients [40]. Our survey findings suggest that the CDS and SDMT may have introduced some uncertainty into this discussion for PCCs given the conflicting USPSTF and institutional recommendations [22], and the different pathways for women with higher risk.

### Limitations

Limitations to this study include those related to self-reported survey research (e.g., social desirability, missing data, and nonresponse bias) [4, 7], as well as the passage of time, the potential impact of other cancer prevention and screening initiatives within the healthcare system, and attrition. Some respondents may also have changed between pre-implementation and post-implementation. While all PCC eligibility criteria were the same for survey recruitments at both time points, PCCs’ individual eligibility may have changed due to changes in care roles and clinics, as well as the addition of new PCCs in clinical practice between pre-intervention and post-intervention surveys. Rooming staff, tasked with printing the CDS handouts for patients and PCCs, may also have changed; however, to address the limitation of changes in PCC and rooming staff, ongoing and multimodal training was provided for all intervention clinic staff by the study team throughout the intervention [1, 2, 8]. Statistical methods comparing pre-implementation and post-implementation responses within study arms assumed independence, although some respondents may have contributed to both sets of survey responses, while others only one. Due to the real-time deidentification of the data, there was no way to determine individual PCC response patterns. Furthermore, individual clinician variance may explain between group differences. Also, analyses were limited to bivariate comparisons. We were unable to compare differences in opinions between PCCs practicing in rural compared to urban clinic locations due to anonymized data. Differences may exist between rural and urban PCCs, as rural healthcare faces a shortage of PCCs and other areas of clinical care [41, 42]. Prior research did explore differences in pre-implementation survey responses by PCC type (advanced practitioners compared to physicians) [4]. Lastly, this survey only gauged PCC opinions. Future research should assess low long shared decision-making conversations between PCCs and patients last when using CDS and/or SDMT for cancer prevention and screening, as well as the quality and outcomes of those discussions. The results of a qualitative study we conducted by interviewing 37 patients seen in 10 of the RCT’s intervention arm clinics immediately after their visits suggested that patients who discussed the CDS with their PCC during their visit may be more likely to make a choice regarding cancer prevention and screening than patients who received the CDS but did not review it with their PCP, a finding that future research should investigate further [6].

## Conclusions

In this pre- and post-implementation survey study of a CDS intervention for cancer prevention and screening in primary care for patients due for breast, cervical, colorectal, or lung cancer screening or HPV vaccination, we found that most PCCs practicing in CDS (with or without SDMT) intervention arms would recommend the CDS to colleagues and found it held value for patients and within their practice. However, PCCs also described lower self-reported CDS use rates than would be expected with these positive perspectives on the primary intervention. Our findings also suggest that the EHR-linked CDS impacted PCC perceptions in areas like printing materials and providing risk calculators. Within intervention arms but not in UC, rates of PCCs’ comfort with discussing breast cancer screening also declined between pre- and post-implementation surveys, suggesting that the CDS may have had an unexpected impact on PCCs level of comfort in this area. Whether reported changes in PCC opinions within study arms would lead to care practice changes, and potential benefits to patients, requires further study. Forthcoming findings from the RCT in which this survey study took place will examine this in more detail, as should future research on cancer prevention and screening CDS with or without SDMT in primary care.

## Abbreviations

ACIP: Advisory Committee on Immunization Practices
CDC: Centers for Disease Control and Prevention
CDS: Clinical decision support
EHR: Electronic health record
HPV: Human papillomavirus
PCC: Primary care clinician
RCT: Randomized control trial
SDMT: Shared decision-making tools
UC: Usual care
USPSTF: United States Preventive Services Task Force
